# Men’s Discomfort and Anticipated Sexual Misclassification Due to Counter-Stereotypical Behaviors: the Interplay between Traditional Masculinity Norms and Perceived Men’s Femininization

**DOI:** 10.1007/s11199-020-01210-5

**Published:** 2020-12-06

**Authors:** Islam Borinca, Vincenzo Iacoviello, Giulia Valsecchi

**Affiliations:** Faculty of Psychology and Educational Sciences, University of Geneva, 40 bd. du Pont d’Arve, CH-1205 Geneva, Switzerland

**Keywords:** Traditional masculinity, Counter-stereotypical behaviors, Male role norms, Discomfort, Social change, Identity misclassification

## Abstract

The present research examines men’s self-conscious discomfort while imagining performing counter-stereotypical (traditionally feminine) behaviors as compared to stereotypical (traditionally masculine) behaviors, as a function of traditional masculinity endorsement and perceived social changes in men’s gender norms (men’s feminization) in two distinct cultural populations (U.S. Americans and Kosovan Albanians). Experiment 1 (*n* = 192, U.S.) confirmed prior findings by showing that men, as compared to women, experienced more self-conscious discomfort when imagining performing counter-stereotypical (vs. stereotypical) behaviors. Experiments 2 (*n* = 292, U.S.) and 3 (*n* = 191, Kosovo) focused solely on heterosexual men and experimentally manipulated perceived social changes in men’s gender norms. Results showed that the men’s feminization condition, as compared to a control or masculinity conditions, decreased self-conscious discomfort when imagining performing feminine behaviors among men who endorsed a lower versus higher level of traditional masculinity. Experiment 3 further indicated that this effect was driven by lowered expectations of being misclassified as gay. We discuss the relevance of these findings for both the gender and sexual prejudice literature.

**Supplementary Information:**

The online version contains supplementary material available at 10.1007/s11199-020-01210-5.

Unlike femininity, masculinity has been depicted as precarious—that is, something hard to win and simple to lose (Bosson and Vandello 2011; Vandello et al. 2008). In addition, the anti-femininity mandate implies that masculinity is predominantly established and maintained in contrast to femininity (Herek 1986; Thompson et al. 1985). Therefore, to demonstrate their masculinity, men need to resist traditional and stereotypical feminine characteristics, roles, and behaviors (Bem 1974; Bosson and Michniewicz 2013; Kilianski 2003; Spence and Helmreich 1979). Indeed, the distinction between masculine and feminine characteristics is more important for men than for women, and this greater gender dichotomization is motivated in particular by the need for men to escape femininity from male gender identity (Bosson and Michniewicz 2013). Because gay men are often perceived as feminine (see Kite and Deaux 1987; Lehavot and Lambert 2007), heterosexual men also need to show their masculinity by avoiding and distancing themselves from homosexuality (Herek 1986; Kite and Deaux 1987; Lehavot and Lambert 2007).

An impressive body of research provides evidence in this regard. From an observer’s point of view, for instance, masculinity is associated with heterosexuality (Herek 1986), as well as powerful, dangerous, and risky behaviors (Brannon and David 1976; Gilmore 1990; Rudman et al. 2012; Schrock and Schwalbe 2009). As a result, a man who enacts feminine behaviors is easily perceived and classified as “not masculine” and “not heterosexual” (Deaux and Lewis 1984; Foushee et al. 1979; McCreary 1994) and then punished in the form of homophobic epithets such as “fag” (Burn 2000; O’Neil and Nadeau 1999; Pryor and Whalen 1997), withdrawal of parental attention and rejection (Fagot 1977; Lamb and Roopnarine 1979; Langlois and Downs 1980; Zheng 2015), and negative evaluations (Feinman 1981; Levy et al. 1995; Sirin et al. 2004). For instance, individuals who saw a heterosexual man interacting with a gay (vs. a straight) person were more likely to negatively assess and condemn him, namely because they deemed him to have gay tendencies and stereotypical feminine traits (Neuberg et al. 1994; Sigelman et al. 1991).

From an actor’s point of view, research indicates that due to the anti-femininity mandate, men are extremely motivated or pressured to embrace traditional masculinity norms and avoid counter-stereotypical behaviors. Younger men in particular feel the most pressure to conform with traditional masculinity norms, with two thirds (67%) of men aged 18–24 feeling compelled to display hyper-masculine behavior, compared to 30% of men over 45 (You Gov 2018). On top of that, men generally report a greater fear of backlash (anxious anticipation of social and economic sanctions) during a gender role violation than do women (Bosson et al. 2006; Rudman and Fairchild 2004). Likewise, men (vs. women) tend to be more concerned with threats to gender status, more reluctant to deviate from ingroup gender norms, and therefore more driven to restore their threatened status (Kosakowska-Berezecka et al. 2016).

Heterosexual men’s motivation not to be misclassified as gay specifically triggers their incentive to avoid female stereotypic behaviors (Bosson et al. 2005, 2006; Rudman and Fairchild 2004; Sirin et al. 2004), and they are thus sensitive to role-violating behaviors likely to lead to such identity misclassification (Bosson et al. 2005, 2006; Herek 1986; Preston and Stanley 1987). Of particular relevance to the present research, men, as compared to women, showed greater self-conscious discomfort in visualizing counter-stereotypic (i.e., feminine) behaviors relative to stereotypic (i.e., masculine) behaviors, mainly due to expectation of identity misclassification (Bosson et al. 2005, 2009a, 2009b). Note that such a self-conscious concern can undermine men’s cognitive and social functioning (Cioffi 2000; Schlenker and Leary 1982) and reduce their well-being (Deci and Ryan 1985; Leary et al. 1995).

Moreover, recurrently disclosing sexual orientation (e.g., public disclaimers) in order to avoid identity misclassification can be extremely demanding, as well as legally or socially sanctioned, and therefore not all men are willing to engage in it (Bosson et al. 2005, 2006; Prewitt-Freilino and Bosson 2008; Stokes and Hewitt 1976). Research also shows that men who endorse the most traditional masculinity norms display stronger preferences for masculine (vs. feminine) careers (Diekman et al. 2011; Tokar and Jome 1998), tend to evaluate more negatively those men who deviate from traditional masculinity norms (i.e., the backlash effect; Glick et al. 2015; Rudman and Glick 2001), and exhibit more negative attitudes toward homosexuality, particularly toward gay men (Blashill and Powlishta 2009; Parrott 2009; Parrott et al. 2011).

Taken together, prior research indicates that men—specifically those men who endorse traditional masculinity norms the most—are strongly motivated to affirm their masculinity and are particularly sensitive to gender norm violations. Specifically, they feel discomfort when they perform counter-stereotypical behaviors because they perceive the risk of being misclassified as gay, and this self-concerns challenges both their cognitive functioning and well-being. The question, therefore, is whether there are factors that may help men experience less self-discomfort while performing counter-stereotypical (feminine) behaviors (see also Bosson et al. 2006); however, there is a lack of evidence in the literature pertaining to this issue. With the present research we aimed to contribute to this effort by examining, for the first time known, the endorsement of traditional masculinity and the role of perceived changes in gender norms—that is, the perception that men are becoming gradually feminine (or not) on self-conscious discomfort when imagining performing counter- stereotypical behaviors and whether the possibility of anticipating misclassification (i.e., expecting to be labeled as gay) would explain such an effect.

## Perceived Men’s Feminization

Despite the current influence of the antifemininity mandate, it seems that there have never been so many ways to be a man, or at least to look like one (Itulua-Abumere 2013; Lomas et al. 2016). For instance, on the red carpet and in magazine pages, it is impossible to ignore the wave of men disregarding stereotypes of masculinity, from “Harry Styles’ outing as the boy with the pearl earring at the 2019 Met Gala to Pharrell Williams donning a down-filled Moncler gown by Valentino on the cover of GQ” (Alleyne 2020, para. 2). People, therefore, may generally perceive that men have become more and more feminine over the past decades (Zafra and Garcia-Retamero 2011, which is consistent with some findings indicating that the presence of men in typically feminine domains is increasing (Champagne et al. 2015; Sani 2014; Scambor et al. 2014). These new trends suggest a new way of being a man that might be perceived as a challenge to traditional masculinity, particularly to the antifemininity norm (cf. Donnelly and Twenge 2017). In the present research, we investigate the potential consequences of these perceived social changes in the antifemininity norm of masculinity (i.e., men’s feminization) on heterosexual men’s self-conscious discomfort when imagining performing counter-stereotypical (feminine) behaviors.

We are not aware of past research investigating this issue, and the few studies examining the consequences of these perceived social changes on different outcomes provided mixed results. On the one hand, some studies showed that providing men with information indicating changes in masculinity norms might relieve them from the need to engage in compensatory mechanisms to rebuild manhood. For instance, priming men to believe that men and women are similar in terms of personality decreased their motivation to justify gender inequalities and increased their willingness to engage in nontraditional parental duties (Kosakowska-Berezecka et al. 2016). On the other hand, past research showed that perceived changes in masculinity norms could reactively motivate men to affirm their masculinity in different ways. As a case in point, men reported a greater likelihood of undertaking different behaviors that broadcast “real man” status to onlookers to varying degrees after being provided with compelling evidence that men are becoming more (vs. less) feminine as a group over time (Bosson and Michniewicz 2013; Study 5). Likewise, men reacted to perceived men’s feminization by affirming alternative masculinity norms, such as heterosexuality and sexual prejudice (Falomir-Pichastor et al. 2019, Study 1).

Nonetheless, past research also suggests that these inconsistent findings can be integrated as a function of men’s initial endorsement of traditional masculinity. Indeed, Babl (1979) demonstrated that men who scored high in masculinity display more negative emotional and behavioral responses when exposed to information representing that men have become more feminine as compared to information indicating that the level of men’s masculinity has not changed (or to a control condition without information about gender). In the same vein, Falomir-Pichastor et al. (2019, Study 2) showed that men who endorsed the most antifemininity norm of masculinity reacted defensively to the perception of men’s feminization by increasing both discomforts toward homosexuality and sexual prejudice. However, a different pattern emerged among men who endorsed masculinity less. In fact, men who scored low in masculinity (i.e., gender-atypical men) did not see the need to restore their manhood when exposed to information indicating men’s change (see Babl 1979). By the same token, men who endorsed the least antifemininity norm of masculinity decreased their discomfort toward sexual minorities in particular when understanding that men are becoming more feminine (Falomir-Pichastor et al. 2019).

## The Present Studies

Despite the relevance of these findings, to our knowledge, no previous research has investigated the effect of perceived social changes in gender norms on men’s self-discomfort while imagining performing feminine behaviors and their expectations of being misclassified as gay. In order to fill in this gap, the present research examined whether men’s endorsement of traditional masculinity norms and perceived men’s feminization jointly influence their self-conscious discomfort when imagining performing counter-stereotypical (feminine), as compared to stereotypical (masculine) behaviors, and whether the expectation of being misclassified as gay would mediate such effect.

We conducted three experiments in two different cultural contexts (United States and Kosovo) in order to investigate this issue. First, we focused on the American context where masculinity tends to be considered precarious and, compared to femininity, is defined as a process that should be related to more social achievements (Vandello et al. 2008). We then focused on the Kosovo context because for the Kosovo-Albanians, masculinity is related to nationalistic or masculine-typed behaviors such as honor, patriotism, courage, and duty (Munn 2008). Therefore, they represent very relevant contexts for testing our hypotheses.

All three of our experiments were conducted in a manner compatible with ethical guidelines for the treatment of human subjects, and our research was approved by the ethical committee of the authors’ home institution. In Experiment 1 (a pilot study), we aimed at confirming that, as compared to women, men feel more discomfort when imagining themselves performing counter-stereotypical behaviors (Bosson et al. 2005). Thus, we asked both male and female heterosexuals to indicate their discomfort while vividly imagining themselves performing either gender counter-stereotypical or stereotypical behaviors.

In Experiments 2 and 3 we focused only on heterosexual men. In Experiment 2 we specifically assessed participants’ endorsement of traditional masculinity and experimentally manipulated gender norms (perceived men’s feminization vs. control). We then compared men’s self-conscious discomfort while imagining themselves performing either counter-stereotypical (feminine) or stereotypical (masculine) behaviors. In Experiment 3, we assessed participants’ endorsement of traditional masculinity again and experimentally manipulated gender norms (perceived men’s feminization vs. masculinity, instead of a control condition). In contrast to previous research, however, we measured their self-conscious discomfort only after they had to visualize themselves performing all counter-stereotypical (feminine) behaviors.

In line with Bosson et al.'s (2005) findings, in Experiment 1, we expected that, as compared to women, men would express more self-conscious discomfort while imagining themselves performing counter-stereotypical (vs. stereotypical) behaviors (Experiment 1; Hypothesis 1). Most relevant for the present purpose, in Experiments 2 and 3 we tested our main hypotheses (Hypotheses 2 and 3). In line with Babl’s (1979) and Falomir-Pichastor et al.'s (2019) findings, we expected that endorsement of traditional masculinity would moderate the effect of gender norm on men’s conscious discomfort while imagining themselves performing counter-stereotypical (feminine) rather than stereotypical (masculine) behaviors (Hypothesis 2). More specifically, in the feminization norm condition (vs. masculine or control conditions), we expected that men who endorse a low level of traditional masculinity would show less self-conscious discomfort when imagining themselves performing feminine behaviors. Regarding men who endorse traditional masculinity more strongly, we expected that the feminization (vs. masculine or control) condition would increase their reported self-conscious discomfort when imagining themselves performing feminine (vs. masculine) behaviors. Finally, Experiment 3 tested Hypothesis 3, according to which the expectation of being misclassified as gay would mediate the effect predicted in Hypothesis 2.

## Experiment 1 (Pilot Study)

Experiment 1 was designed to replicate Bosson et al. ‘s (2005) findings, according to which, as compared to women, men would feel more self-conscious discomfort when imagining performing feminine (counter-stereotypically) than masculine (stereotypically) behaviors.

### Method

#### Sample and Procedure

Following guidelines by Simmons et al. ‘s (2013), we determined a priori the need to recruit at least 50 participants per experimental condition (see also Nook et al. 2016). Hence**,** given our 2 × 2 design, we recruited 222 U.S. participants online via MTurk, but we excluded data from 30 of them, who were not self-classified as heterosexual. As a result, the final sample consisted of 192 heterosexual participants (81 heterosexual women) (*M*_age_ = 37.54, *SD*_age_ = 11.61, *mdn* = 33, range = 20–69), and with no age difference between women and men, *t*(190) = −1.70, *p* = .089. Participants were randomly assigned to one of the four conditions in a 2 (Gender: male vs. female) × 2 (Behavior type: feminine vs. masculine) between-subjects design. In all experiments, participants were carefully debriefed and thanked. A sensitivity analysis considering ANOVA (main effects and the interaction term), assuming an alpha of .05 and power of .80, revealed that our final sample size was enough to detect an effect size of *f* = .20 (i.e., a small effect size; Faul et al. 2009).

#### Behavior Type

Depending on the experimental condition, participants were invited to vividly imagine performing either six stereotypically feminine behaviors (i.e., dancing in a ballet class, styling someone’s hair, designing a living room, shopping for clothes with several female friends, reading a fashion magazine, and talking to friends about emotions) or six stereotypically masculine behaviors (i.e., working on a construction project, doing an engineering project, doing a strength-training workout, hunting, treating patients in a medical practice, and watching a football game with friends; see Bosson et al. 2005). In each condition, these behaviors were presented in the same afore-mentioned order.

#### Self-Conscious Discomfort

Immediately after visualizing themselves performing each behavior, participants answered seven items also adapted from Bosson et al. (2005) such as “How uncomfortable would you feel?”; “How embarrassed would you feel?”; and “How concerned would you be about the impression you would make on others?” Responses were reported on a 7-point Likert-type scale from 1 (*Not at all*) to 7 (*Absolutely*) (see also Prewitt-Freilin and Bosson 2008). However, and in order to simplify the procedure, participants responded to these items regarding the six behaviors together instead of each behavior separately. We computed an average score of self-conscious discomfort such that higher scores indicated stronger discomfort (α = .76; *M* = 3.56, *SD* = 1.86).

### Results

We performed a full-factorial 2 × 2 ANOVA on self-conscious discomfort by including participant’s gender (male vs. female) and behavior type (feminine vs. masculine) as independent variables. Results showed a significant main effect of gender, *F*(1, 188) = 6.91, *p* = .009, η_p_^2^ = .03, with men feeling overall more self-conscious discomfort (*M* = 3.74, *SD* = 1.44, range = 1–7) than women (*M* = 3.23, *SD* = 1.41, range = 1–6.7). The main effect of behavior type was also significant, *F*(1, 188) = 5.66, *p* = .018, η_p_^2^ = .02, with more self-conscious discomfort expressed in the feminine behavior condition (*M* = 3.87, *SD* = 1.41) than in the masculine behavior condition, (*M* = 3.21 *SD* = 1.40).

Importantly, the participants’ gender x behavior type interaction was significant, *F*(1, 188) = 20.50, *p* < .001, η_p_^2^ = .09. Planned comparisons showed that in the feminine behavior type condition, men (*M* = 4.39, *SD* = 1.75) expressed higher level of self- conscious discomfort than did women (*M* = 2.99, *SD* = .22), *F*(1, 188) = 23.82, *p* < .001, η_p_^2^ = .12, whereas no gender discrepancy appeared in the masculine behavior type condition (*M*_men_ = 3.04, *SD* = .18; *M*_women_ = 3.41, *SD* = .19), *F*(1, 188) = 1.95, *p* = .164, η_p_^2^ = .01. Additional analyses indicated that men expressed higher level of self-discomfort in the feminine behavior type than in masculine behavior type condition, *F*(1, 188) = 28.67, *p* < .001, η_p_^2^ = .13, whereas this effect was not significant among women, *F*(1, 188) = 1.97, *p* = .161, η_p_^2^ = .01.

### Discussion

Consistent with Bosson et al.'s (2005) findings, Experiment 1 provided evidence in support of Hypothesis 1. U.S. men reported high levels of self-discomfort when they envisioned themselves performing counter-stereotypical (feminine) rather than stereotypical (masculine) behaviors, whereas a similar pattern was not observed for U.S. women. Given that visualizing oneself doing counter-stereotypical behaviors triggered discomfort among men, but not among women, in the following experiments, we focused only on men in order to test our main hypotheses (Hypotheses 2 and 3).

## Experiment 2

In Experiment 2 we considered only a sample of heterosexual men. We initially measured endorsement of traditional masculinity norm as an individual difference and then experimentally manipulated gender norm (perceived men’s feminization vs. control). Finally, we experimentally manipulated behavior type (feminine vs. masculine) as in Experiment 1. According to Hypothesis 2, we expected that participants who endorse a low level of traditional masculinity would feel more at ease with themselves when imagining performing counter-stereotypical behavior in the men’s feminization condition, as compared to the control condition. However, the men’s feminization condition should increase self-conscious concerns when imagining performing feminine behavior among participants who more strongly endorse traditional masculinity.

### Method

#### Sample and Procedure

Because we had a 2 × 2 experimental design again, but we additionally assessed endorsement of masculinity norm as an individual difference (a continuous variable), we decided to increase the sample size of the present experiment as compared to Experiment 1. Therefore, we initially recruited 322 U.S. men via MTurk. After the data inspection, 28 participants were not self-classified as heterosexual and two were self-classified as female and thus these participants were excluded from the data analysis.

The final sample consisted of 292 heterosexual men (*M*_age_ = 39.79, *SD* = 12.47, *mdn* = 37, range = 18–78). We initially assessed participants’ endorsement of traditional masculinity and then randomly assigned them to one condition in a 2 (gender norm: control vs. feminization) × 2 (behavior type: masculine vs. feminine) experimental design. The sensitivity analysis considering a multiple linear regression model with seven predictors (3 main effects, 3 two-way interactions and 1 three-way interaction), assuming an alpha of .05 and power of .80, revealed that our final sample size was powered enough to detect an effect size of *f* ^*2*^ = .05 (which, by convention indicates a small effect size; see Faul et al. 2009).

#### Endorsement of Traditional Masculinity

Participants’ endorsement of traditional masculinity norms was assessed through the 26-item Male Role Norms Scale (MRNS; Thompson and Pleck 1987). Sample items are: “I might find it a bit silly or embarrassing if a male friend of mine cried over a sad love scene in a movie”; “When a man is feeling a little pain he should try not to let it show very much”; and “A man should never back down in the face of trouble.” Response scales ranged from 1 (*not at all*) to 7 (*absolutely*). We computed an average overall score so that higher scores reflected greater endorsement of traditional masculinity (α = .93; *M* = 3.82, *SD* = 1.11).

#### Men’s Gender Norm

We then experimentally manipulated gender norms by exposing participants to a supposedly “real” scientific article concerning men’s gender evolution. In the men’s feminization condition, the article indicated that men are changing and becoming more feminine. The material was adapted from Falomir-Pichastor et al. (2019), and presented as a scientific research report, including some pictures in which men were depicted as performing feminine behaviors or wearing female clothes (see the online supplement). We compared this condition to a control condition in which participants did not receive any information concerning men’s gender evolution.

#### Behavior Type and Self-Conscious Discomfort

As in Experiment 1, participants were invited to visualize themselves performing either six typically feminine behaviors or six typically masculine behaviors. We used the same items as in Experiment 1 to assess participants’ self-discomfort (α = .90; *M* = 3.26, *SD* = 1.52).

#### Manipulation Check

We used four items asking participants to indicate their opinion concerning men’s evaluation: “Men’s behavior has changed in recent years”; “Men’s way of being has changed in recent years”; “Today, men are more feminine than ever”; and “Today, men are still as masculine as they were in the past” (reversed). Scales ranged from 1 (*not at all*) to 7 (*absolutely*). An overall score was computed by averaging the response to these four items, wherein higher scores reflect an acknowledgment of men’s feminization (α = .87; *M* = 4.69, *SD* = 1.37).

### Results

We regressed all dependent variables on the endorsement of traditional masculinity (standardized scores), gender norm (control vs. feminization), and behavior type (masculine vs. feminine), as well as all interactions between these three factors.

#### Manipulation Checks

The regression analysis revealed a main effect of participants’ endorsement of traditional masculinity: Greater endorsement of masculinity was related to a greater perception of men’s feminization (*B* = .35, *SE* = .08), *t*(283) = −1.35, *p* < .001, 95% CI = [.19, .51], *d* = .50. In addition, we observed a significant main effect of the gender norm manipulation, *t*(283) = 2.27, *p* = .024, 95% CI = [.02, .33], *d* = .27. Participants perceived that men are becoming more feminine in the feminization condition (*M* = 4.88, *SD* = 1.39; range = 1–7) than in the control condition (*M* = 4.53, *SD* = 1.33; range = 1–7). No other effect was significant.

#### Self-Conscious Discomfort

The regression analysis showed a main effect of participants’ endorsement of traditional masculinity: Greater endorsement of masculinity was related to more self-conscious discomfort (*B* = .42, *SE* = .07), *t*(283) = 4.96, *p* < .001, 95% CI = [.28, .56], *d* = .70. The main effect of behavior type was also significant, *t*(283) = −10.95, *p* < .001, 95% CI = [−.89, −.62], *d* = 1.30, and was qualified by the two-way interaction between behavior type x endorsement of masculinity (*B* = −.51, *SE* = .07), *t*(283) = −7.09, *p* < .001, 95% CI = [−.65,. -.36], *d* = .84, and by the expected gender norm x endorsement of masculinity x behavior type three-way interaction (*B* = −.15, *SE* = .07), *t*(283) = −2.12, *p* = .034, 95% CI = [−.29, −.011], *d* = .25.

**Fig. 1:**
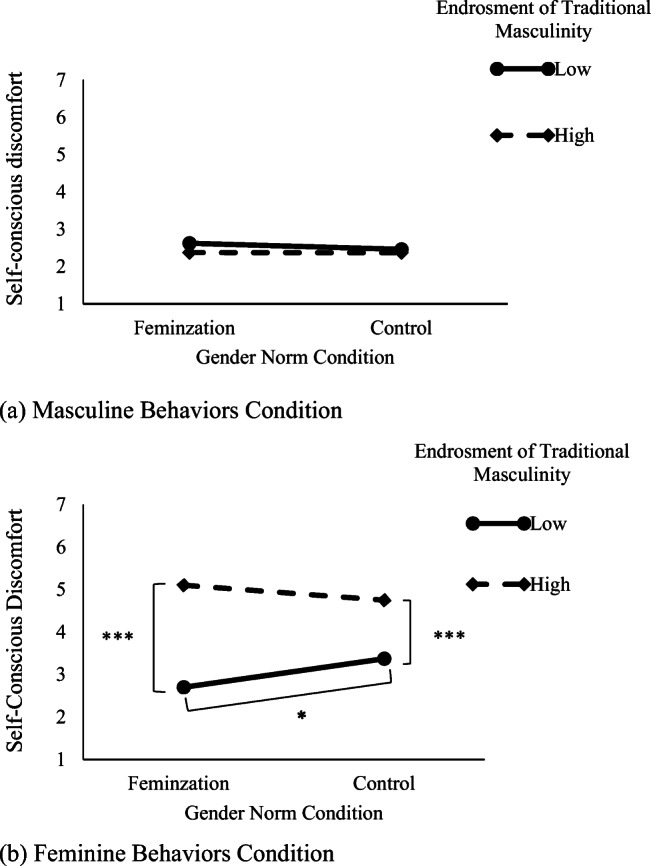
Men’s self-conscious discomfort while imagining themselves performing either (**a)** feminine or (**b**) masculine type behaviors as a function of the gender norm condition (feminization wherein men are being feminized vs. no-information control) to which they were exposed and their endorsement of traditional masculinity at low (−1 SD) and high (+1 SD) levels, experiment 2. *p < .05. ***p < .001. (**a**) Masculine behaviors condition, (**b**) feminine behaviors condition

Exploring the three-way interaction, the two-way interaction between gender norm condition x endorsement of masculinity in the masculine behavior condition was not significant, (*B* = −.04, *SE* = .09), *t*(283) = −.42, *p* = .671, 95% CI = [−.22, .14], *d* = .06 (see Fig. 1a). Regarding the feminine behavior condition, the main effect of endorsement of masculinity was significant (*B* = .936, *SE* = .10), *t*(283) = 8.60, *p* < .001, 95% CI = [.72, .15], *d* = .20. More importantly, in the feminine behavior condition, the interaction between gender norm condition x endorsement of masculinity was significant (*B* = .26, *SE* = .10), *t*(283) = 2.44, *p* = .015, 95% CI = [.05, .47], *d* = .29. The decomposition of this interaction indicated that men who endorsed masculinity at lower levels (−1 *SD*) showed less discomfort in the feminization condition than in the control condition, *t*(283) = −2.21, *p* = .027, 95% CI = [−1.26, −.07], *d* = .26, but this difference was not significant among those men who more strongly endorsed traditional masculinity (+1 *SD*), *t*(283) = 1.38, *p* = .166, 95% CI = [−.16, .94], *d* = .16 (see Fig. 1b). Additional analyses within the feminine behaviors condition showed that endorsement of traditional masculinity increased participants discomfort both in the feminization norm condition, (*B* = 1.20, *SE* = .17), *t*(283) = 6.69, *p* < .001, 95% CI = [.84, 1.55], *d* = .79, and in the control condition, (*B* = .67, *SE* = .12), *t*(283) = 5.45, *p* < .001, 95% CI = [.42, .91], *d* = .64, but this effect was stronger in the feminization norm condition.

### Discussion

The results of Experiment 2 provided evidence in support of Hypothesis 2, according to which participants’ endorsement of masculinity moderated the effect of gender norm. More specifically, participants who endorsed masculinity at a lower level felt less discomfort while visualizing themselves performing feminine behavior in the feminization condition compared to in the control condition. However, unexpectedly, no significant findings were observed among participants who more strongly endorsed masculinity. Before concluding, we conducted a third and final experiment in order to further test and increase the generalizability of our findings.

## Experiment 3

As compared to Experiment 2, in Experiment 3 we introduced four main changes. First, past research has shown that the social context can influence people’s understanding of gender norms and their inclination to deviate from them (Kosakowska-Berezecka et al. 2016). Therefore, in the present experiment we used a culturally different sample—that is, Kosovan Albanians. Second and most importantly, we assessed the likelihood of being misclassified as gay in order to examine whether this expectation mediates the effect of the interplay between gender norm and endorsement of traditional masculinity on men’s self-conscious discomfort (Hypothesis 3). Third and for simplicity reasons, we focused exclusively on counter-stereotypical (feminine) behaviors. Finally, we modified the content of the gender norm manipulation and compared the feminization condition to a masculine norm condition instead of the control (no information) condition we used in Experiment 2.

### Method

#### Sample and Procedure

Recruitment took place on the vast campus of a Kosovo university and various public areas around. Kosovan-Albanian men were voluntarily invited to fill in a paper-and-pencil questionnaire on the way people perceive different groups. Given that this experiment included only two experimental conditions and a continuous predictor, we used a 2 × 2 design as a proxy to determine the sample size. Following Simmons et al. ‘s (2013) guidelines, we recruited a total of 200 Kosovan Albanian men who voluntarily agreed to participate in the experiment. Of these, nine participants were not included in the final analyses because they did not self-identify as heterosexuals.

The final sample consisted of 191 heterosexual men (*M*_age_ = 25.40, *SD* = 5.82, *mdn* = 24, range = 18–65). We adopted a quasi-experimental approach in which we initially assessed participants’ endorsement of traditional masculinity and then randomly assigned them to one of the two experimental conditions of men’s gender norm (masculine vs. feminization). All participants were then invited to vividly visualize themselves performing the six feminine behaviors used in previous experiments. The sensitivity analysis considering a multiple linear regression model with three predictors (two main effects and their interaction), assuming an alpha of .05, and power of .80, revealed that our final sample size was powered enough to detect an effect size of *f* ^*2*^ = .05 (which, by convention indicates a small effect size; see Faul et al. 2009). It is worth noting that all the materials for this experiment have been translated and adapted to the Albanian language.

#### Endorsement of Traditional Masculinity

As in Experiment 1, we used the Male Role Norms Scale (Thompson and Pleck 1987) to assess participants’ endorsement of traditional masculinity norms (α = .76; *M* = 4.26, *SD* = .75).

#### Men’s Gender Norm

In order to provide consistent support to our hypotheses while using different experimental materials, in the present experiment we changed the content of the norm manipulation and the comparison condition. Participants were randomly assigned to one of two experimental conditions (feminization vs. masculine), also adapted from Falomir-Pichastor et al. (2019; Study 1). In both conditions, participants read a one-page text (ostensibly published in a scientific journal of sociology) summarizing the results of an international study about the evolution of men’s gender identity in society. Participants were informed that this study was conducted between 1990 and 2010 on a representative sample of the population in Western countries, and it assessed all relevant criteria that are traditionally recognized as distinguishing masculinity from femininity (such as physical appearance concerns, emotionality, sensitivity, investment in housework, romantic relationships and family, childcare, and the importance of one’s career; see the online supplement for the Albanian version).

These fictitious results were summarized in a figure representing the evolution of men’s gender norms on a continuum ranging from masculinity to femininity endpoints. In the *masculine norm condition*, the results stated that “men’s masculinity is stable: men remain clearly masculine and distinct from women. The distinction between masculinity (‘being a man’) and femininity (‘being a woman’) remains fundamental.” In the *feminization norm condition*, the results stated that “men’s masculinity is changing: there is a real ‘feminization of men.’ The distinction between masculinity (‘being a man’) and femininity (‘being a woman’) tends to disappear.”

#### Manipulation Check

After the experimental manipulation, participants in the masculine and feminization conditions indicated the extent to which the study’s conclusions were that: “Men’s behavior has changed in recent years”; “Men’s way of being has changed in recent years”; “Today, men are more feminine than ever”; and “Today, men are still masculine as they were in the past “ (reverse scored), rated from 1 (*not at all*) to 7 (*absolutely*). An overall score was computed by averaging the response to these four items, wherein higher scores reflect an acknowledgment of men’s feminization (α = .61; *M* = 4.69, *SD* = 1.23).

#### Feminine Behavior Type and Self-Conscious Discomfort

All participants were then required to vividly imagine themselves performing the same six feminine behaviors used in the previous experiments. For length limitations related to recruitment conditions and the use of a paper-and-pencil questionnaire, in the present experiment we only retained three items from Bosson et al. (2005) in order to measure participants’ self-discomfort: “How uncomfortable would you feel?”; “How embarrassed would you feel?”; and “How concerned would you be about the impression you would make on others?,” rated form 1(*Not at all*) to 7 (*Absolutely*). We computed an average score of self-concerns such that higher scores indicate greater self-conscious discomfort (α = .62; *M* = 3.88, *SD* = 1.49).

#### Likelihood of Being Misclassified as Gay

Finally, the likelihood of being misclassified as gay was assessed as in Bosson et al.'s (2005, 2006) research, but we included two additional items (see the online supplement for the Albanian version). Thus, participants answered three items assessing the likelihood of being misclassified by people who did not know them while performing the feminine behaviors: “The likelihood that someone who did not know you would automatically assume that you were gay if he/she saw you performing each of the behaviors?”; “The likelihood that a heterosexual person would automatically assume that you were gay too if he/she saw you performing each of the behaviors?”; and “The likelihood that a woman would automatically assume that you were gay too if he/she saw you performing each of the behaviors?,” rated from 1(*Very unlikely*) to 7 (*Very likely*) (α = .90; *M* = 3.66, *SD* = 1.97). An overall score was calculated by averaging across items such that higher scores indicate a greater perceived likelihood of being misclassified as gay.

### Results

We regressed all dependent variables on the endorsement of traditional masculinity (standardized scores), gender norm (feminization vs. masculine), and the interaction between these two variables.

#### Manipulation Checks

The regression analysis revealed a main effect of participants’ endorsement of traditional masculinity: Greater endorsement of masculinity was related to greater perception of men’s feminization (*B* = .25, *SE* = .08), *t*(187) = 2.89, *p* = .004, 95% CI = [.08, .42], *d* = .42. In addition, we observed a significant main effect of the gender norm manipulation, *t*(187) = −2.10, *p* = .037, 95% CI = [−.35, −.01], *d* = .30. Participants acknowledged that men are becoming feminine over time in the feminization condition (*M* = 4.92, *SD* = 1.08, range = 2.5–7) than in the masculine condition (*M* = 4.52, *SD* = 1.32, range = 1–7). No other effect was significant (*p*s > .150).

#### Self-Conscious Discomfort

**Fig. 2:**
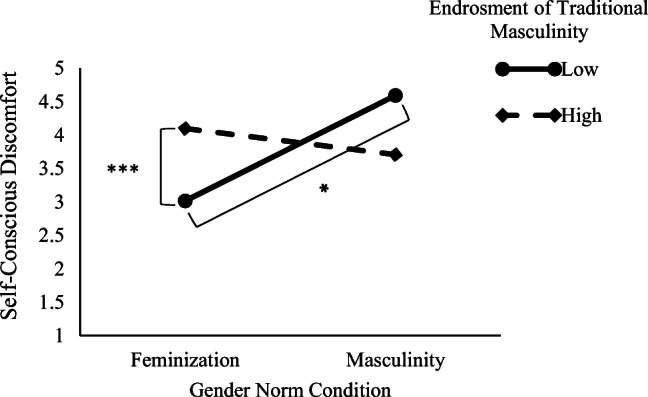
Men’s self-conscious discomfort while imagining themselves performing feminine type behaviors as a function of their gender norm condition (feminization wherein men are being feminized vs. masculinity is stable) to which they were exposed and their low (−1 *SD*) or high (+1 *SD*) endorsement of traditional masculinity, experiment 3. *p < .05. ***p < .001

Results showed a main effect of participants’ endorsement of masculinity: Greater endorsement of masculinity was related to greater self-concerns (*B* = .50, *SE* = .10), *t*(187) = 4.96, *p* < .001, 95% CI = [.30, .70], *d* = .72. Further, the gender norm x participant’s endorsement of masculinity interaction effect was significant (*B* = −.28, *SE* = .10), *t*(187) = −2.78, *p* = .006, 95% CI = [−.48, −.08], *d* = .40 (see Fig. 2). The decomposition of this interaction indicated that men who endorsed traditional masculinity less (−1 *SD*) showed lower self-conscious discomfort in the feminization condition than in the masculine condition, *t*(187) = 2.38, *p* = .018, 95% CI = [.11, 1.25], *d* = .34. However, this effect was not significant among those who more strongly (+1 *SD*) endorsed masculinity, *t*(187) = −1.56, *p* = .118, 95% CI = [−1.01, .11], *d* = .22. Additional analysis indicated that in the feminization norm condition, the less participants endorsed traditional masculinity, the less they expressed self-conscious discomfort (*B* = .78, SE = .14), *t*(187) =5.29, *p* < .001, 95% CI = [.49, 1.08], *d* = .77. However, this effect was not significant in the masculine norm condition (*B* = .22 = .13), *t*(187) = 1.59, *p* = .113, 95% CI = [−.05, .49], *d* = .22.

#### Likelihood of Being Misclassified

**Fig. 3:**
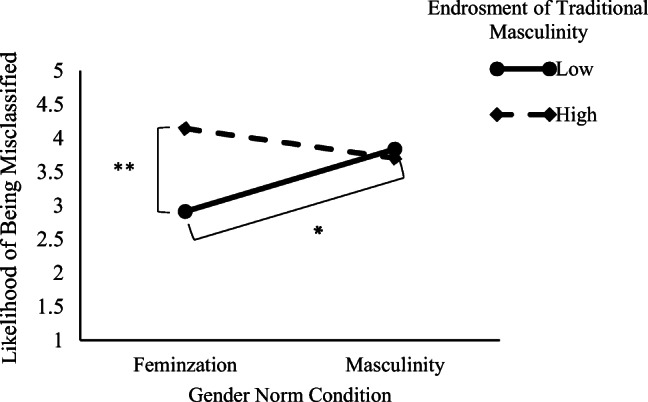
Heterosexual men’s likelihood of being misclassified as gay while imagining themselves performing feminine type behaviors as a function of their gender norm condition (feminization wherein men are being feminized vs. masculinity is stable) to which they were exposed and their low (−1 *SD*) or high (+1 *SD*) endorsement of traditional masculinity, experiment 3. *p < .05. **p < .01

The same regression analysis on the expectation of being misclassified as gay showed a main effect of participants’ endorsement of masculinity: Greater endorsement of masculinity was related to a greater expectation of being misclassified as gay (*B* = .26, *SE* = .14), *t*(187) = 1.86, *p* = .064, 95% CI = [−.01, .54], *d* = .20. The gender norm x participant’s endorsement of masculinity interaction effect was also significant (*B* = −.32, *SE* = .14), *t*(187) = −2.31, *p* = .022, 95% CI = [−.60, −.04], *d* = .28 (see Fig. 3). The decomposition of this interaction indicated that, men who endorsed traditional masculinity lower (−1 *SD*) expected being misclassified as gay to a lesser extent in the feminization condition than in the masculine condition, *t*(187) = 2.30, *p* = .022, 95% CI = [.13, 1.71], *d* = .33. However, this effect was not significant among those who more strongly (+1*SD*) endorsed masculinity, *t*(187) = −.97, *p* = .329, 95% CI = [−1.17, .39], *d* = .14. Additional analysis revealed that in the feminization norm condition, the less participants endorsed traditional masculinity, the less they expected to be misclassified as gay (*B* = .59, SE = .20), *t*(187) = 2.85, *p* = .005, 95% CI = [.18, 1.00], *d* = .41. However, this effect was not significant in the masculine norm condition (*B* = −.06, SE = .19), *t*(187) = −.33, *p* = .738, 95% CI = [−.44, .31], *d* = .06.

#### Mediation Analysis

**Fig. 4:**
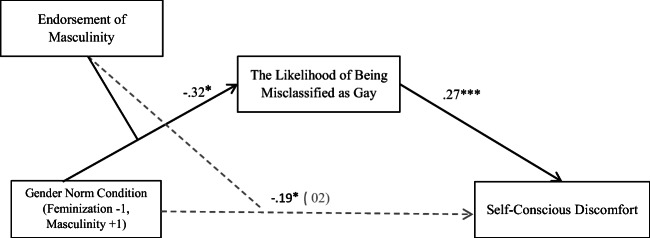
Standardized regression weights and indirect effects for the moderated mediation model in which the effect of gender norm condition (feminization wherein men are being feminized vs. masculinity is stable) on self-conscious discomfort is moderated by endorsement of masculinity, which is then mediated by the expectation of being misclassified as gay, experiment 3. The direct effect of gender norms on self-conscious discomfort is in parentheses. *p < .05. ***p < .001

In order to test Hypothesis 3, we ran a mediation analysis using PROCESS for SPSS (Model 8; Hayes 2018; 5000 bootstrapped samples), in which gender norm condition was the predictor, endorsement of masculinity was the moderator, and self-conscious discomfort was the dependent variable. Additionally, the likelihood of being misclassified as gay was entered as the mediator (see Fig. 4). The analysis showed that the indirect effect of gender norm through expected misclassification was significant among men who endorsed masculinity less (*B* = .12, bootstrapped *SE* = .05, 95% CI [.01, .24]), but not among those who endorsed masculinity more strongly (*B* = −.04, bootstrapped *SE* = .05, 95% CI [−.16, .06]. Moreover, the moderated mediation index was significant, yielding a value that did not include 0 in the confidence interval (Index = −.09, 95% CI [−.18, −.01]).

### Discussion

The results of Experiment 3 were consistent with those observed in Experiment 2 and provided further evidence in support of Hypothesis 2. Men who endorse a low level of traditional masculinity felt less discomfort while imagining performing feminine behaviors in the feminization norm condition as compared to the masculine norm condition. However, as in Experiment 2, the difference between the two experimental conditions was not significant among men who more strongly endorsed traditional masculinity. Finally, Experiment 3, for the first-time, provided evidence in support of Hypothesis 3, according to which the investigated effect on self-conscious discomfort is mediated by the likelihood of being misclassified as gay. More specifically, in the feminization norm condition, as compared to the masculinity norm condition, participants who endorsed a low level of traditional masculinity felt less discomfort while imagining performing feminine behaviors, and this pattern emerged because these participants perceived a lower likelihood of being misclassified as gay. However, the mediation analysis was not significant among men who more strongly endorsed traditional masculinity.

## General Discussion

The present research showed for the first time known that endorsement of traditional masculinity and perceived men’s feminization combined to influence men’s self-conscious discomfort when imagining performing counter-stereotypical (feminine), as compared to stereotypical (masculine), behaviors. Experiment 1 demonstrated that U.S. heterosexual men, as compared to U.S. heterosexual women, feel greater self-conscious discomfort when imagining performing counter- (vs. non-counter) stereotypical behaviors. Two further experiments were conducted in order to better understand the conditions under which men could feel less discomfort when imagining performing counter-stereotypical behaviors. In two culturally different samples, Experiments 2 (U.S. Americans) and 3 (Kosovo Albanians) showed that men who endorsed masculinity at lower levels reported less self-conscious discomfort in the social change condition (feminization) as compared to other conditions (control [in Experiment 2] or hegemonic [in Experiment 3]) when imagining performing feminine behaviors. Finally, Experiment 3 also showed that the expectation of being misclassified as gay explained the effect of gender norm condition on the main dependent variable (self-conscious discomfort). These results, however, were not observed among men who endorsed traditional masculinity more strongly.

Taken together, the present findings may be of relevance regarding research on men’s reactions pertaining to traditional masculinity norm violations. Indeed, prior research has shown that heterosexual men, and especially those who endorse masculinity to a greater extent, show a defensive reaction by reasserting their status as men (Diekman et al. 2011; Tokar and Jome 1998) and by punishing those men who deviate from traditional masculinity (i.e., the backlash effect; Blashill and Powlishta 2009; Glick et al. 2015; Parrott et al. 2011; Rudman and Glick 2001). In addition, heterosexual men (vs. women) feel uneasy when performing counter-stereotypical behaviors, which appears to be mainly because of heterosexual men’s expectation of being misclassified as gay (Bosson et al. 2006, 2008; Rudman and Fairchild 2004). The present research used information on the general feminization of men (rather than the opportunity to declare their heterosexuality) to mollify heterosexual men and ease their concerns about appearing gay and therefore showed that the reactions of heterosexual men to their own violations of traditional masculinity norms rely on their endorsement of traditional masculinity and perceived social change in the norm. Finally in Experiment 3, these findings from the United States were replicated and extended in a different cultural context (i.e., Kosovo).

On the one hand, the present findings showed that men who endorse traditional masculinity at lower levels felt less discomfort when imagining performing feminine behaviors; this effect was enhanced as a function of perceived social changes in the antifemininity norm of masculinity (i.e., men’s feminization). Furthermore, this effect explicitly appeared in this condition because men assumed that they were less likely to be subject to identity misclassification (i.e., being misclassified as gay). Our findings are thus consistent with research showing that men might more easily engage in feminine behaviors when their concerns about being misclassified as gay are surmounted. To explain that dynamic, people who consistently transgress traditional gender roles—such as men in female stereotypic occupations (Williams 1989)—are more likely to feel less self-discomfort because they have built up self-protective immunity to threats associated with identity misclassification (cf. Crocker and Major 1989). Indeed, Bosson et al. (2006) argued that heterosexual adult gender role violators might harbor fewer expectations of identity misclassification than adolescents, given that marriage and parenthood can serve as indirect “proof” of heterosexual status. Therefore, the perception that men are becoming more and more feminine provides heterosexual men who endorse traditional masculinity at low levels with sufficient normative conditions, allowing them to engage in feminine behaviors without feeling discomfort.

On the other hand, it is noteworthy that no significant differences were observed among those men who endorsed traditional masculinity more strongly. Indeed, the present findings showed that heterosexual men who endorsed traditional masculinity more strongly felt discomfort to a greater extent when imagining performing feminine behaviors, but this effect was not influenced by their perception of men’s feminization. This result might seem at odds with past research showing more defensive reactions among these men. Indeed, heterosexual men high in traditional masculinity experience negative feelings (anxiety), report increased levels of masculinity, and show antisocial behavior (Babl 1979) and more negative attitudes toward sexual minorities (Falomir-Pichastor et al. 2019), specifically when they perceive that that masculinity is changing—that is, when men as a group are becoming more and more feminine. Instead, the present findings suggest that men’s perceived feminization may lead to different defensive reactions among men who more strongly support traditional masculinity; future research is needed to further investigate this issue.

Different explanations could be advanced for the lack of significant findings among participants who endorsed masculinity more strongly (i.e., relatively traditional men). For instance, traditional men might internalize a traditional masculinity norm and feel greater discomfort with themselves when imagining performing feminine behaviors, despite perceived social changes (i.e., feminization). Another possibility is that some traditional men might conform to the new femininization norm (i.e., decreasing self-concerns about counter-normative behavior), whereas others reveal a defensive reaction to this norm (i.e., increased self-concern about counter-normative behavior). Given this speculation, both processes could work at the same time and, consequently, may cancel each other in group-level data.

Another possibility is that traditional men might still feel discomfort while imagining performing feminine behaviors, regardless of perceived changes in the antifemininity norm of masculinity; indeed, this result was observed in the current research. However, men’s perceived feminization may motivate them to affirm alternative norms of masculinity, such as heterosexuality and sexual prejudice (see Falomir-Pichastor et al. 2019). Relatedly, Bosson et al. (2006) argued that men experience discomfort when performing feminine behaviors, irrespective of whether they have internalized traditional masculinity norms or specific self-views (e.g., masculinity and femininity), merely because they are conscious of the likelihood of being misclassified as gay. This reasoning is consistent with research showing that men who perceive themselves as masculine, along with men who indirectly assert their masculinity, experience fewer adverse affective reactions when performing counter stereotypical behaviors (i.e., a public hairstyling task; see Prewitt-Freilino and Bosson 2008). Therefore, in the present research, traditional men in the men’s feminization condition did not increase their level of discomfort while imagining performing feminine behaviors because their defensive reaction may have been oriented toward the affirmation of masculinity by other means such as their heterosexuality. In order to better understand whether and why traditional men respond defensively to the perceived feminization of men, future research is needed.

### Limitations and Future Research Directions

Despite the theoretical importance of our results, a few limitations of the present research need to be underlined. To begin with, prior research has shown that women can also experience some level of self-conscious discomfort with some particular behaviors (e.g., attending a peace rally or joining a computer programming group), namely because they expect identity misclassification (Bosson et al. 2005; Study 2; see also Bosson et al. 2006). Because we included only male participants in Experiments 2 and 3, future research should investigate whether endorsement of feminine gender norms and perceived social changes in these norms could also result in a similar pattern of findings among female participants.

Another limitation of our studies relates to the fact that we only asked participants to vividly envision themselves performing feminine versus masculine behaviors, but never placed them in such circumstances. It is worth noting that past research has shown that heterosexual men also experience self-conscious discomfort when performing real feminine behaviors (e.g., hairstyling vs. a rope reinforcing task; Bosson et al. 2005; Study 3; see also Prewitt-Freilino and Bosson 2008). Thus, future research should examine whether the combined effect of endorsement of masculinity and perceived men’s feminization on self-conscious discomfort also appears concerning the actual performance of stereotypical and counter-stereotypical behaviors.

Finally, we should note that our research examined two distinct contexts in terms of cultural values: Kosovo, which represents a collective society, and the United States, which represents an individualistic society. Therefore, future researchers should examine whether cultural values (e.g., living in an individualistic vs. collective society) play a role in how men endorse traditional masculinity norms and experience discomfort when imagining performing feminine behaviors by comparing these cultural values within the same experimental design (for example, see Cuddy et al. 2015).

### Practice Implications

Women, as compared to men, appear more open to imagining performing counter-stereotypical behaviors. Moreover, men who endorse traditional masculinity at lower levels seem to be more at ease with themselves while imagining performing counter-stereotypical behaviors, but only when they realize that social change prevails (i.e., it is acceptable for men to do household chores or take care of children). Then the first question is how we can encourage these men to experience less discomfort when imagining performing counter-stereotypical behaviors, even when the norm does not imply social change, but rather traditional masculinity? The second question is how can encourage men who more strongly endorse traditional masculinity to imagine themselves performing feminine behaviors? Finally, how is all of this related to expectations of being misclassified as gay?

Practitioners (e.g., policymakers, therapists, psychologists, teachers, activists) will need to work together to unravel the connection between the behaviors (masculine vs. feminine) and perceived sexual orientation (e.g., being perceived as gay or heterosexual) to which men appear to give importance. In other words, men need to understand that imagining performing certain feminine behaviors in front of others does not make them appear gay. In fact, even gay men can perform both feminine and masculine behaviors. Understanding the reasons why men tend to connect feminine behaviors to the expectation of sexual misclassification not only would benefit them at an interpersonal level (e.g., experience less discomfort) but also would enhance intergroup relations between gay and heterosexual people. Possibly this outcome could be achieved through different interventions at both individual and collective levels.

### Conclusion

It is important to encourage men to imagine themselves performing counter-stereotypical behaviors without experiencing discomfort for at least two reasons. First, some situations are felt to require a feminine-stereotyped response, whereas others are instead perceived as requiring a masculine-stereotyped response. Individuals who are able to respond flexibly to such situational characteristics are therefore seen as superior in social skills, better suited to the social environment, and ultimately better situated to achieve positive mental health (Annandale and Hunt 1990). This interpretation builds on the earlier gender schema theory of Bem (1981), according to which the integration of male and female traits enables an individual to be a fully functional and adaptive human being. Second, performing feminine behaviors without feeling discomfort allows men to alleviate and protect themselves from detrimental health consequences. Therefore, our findings address the potential benefits of social changes in traditional masculinity norms (at least among those men who do not strongly endorse traditional masculinity norms) among heterosexual men imagining performing counter-stereotypical behaviors and avoiding expectations of being misclassified as gay.

## Supplementary Information

ESM 1 (DOCX 539 kb)
