# Supplementary material for: Men’s Discomfort and Anticipated Sexual Misclassification Due to Counter-Stereotypical Behaviors: the Interplay between Traditional Masculinity Norms and Perceived Men’s Femininization
Source: Sex Roles. 2020 Dec 6;85(3-4):128–41. doi: 10.1007/s11199-020-01210-5 (PMC8550511; doi:10.1007/s11199-020-01210-5)
Supplement: Supplementary file 1 — (DOCX 539 kb) [file 11199_2020_1210_MOESM1_ESM.docx]

Online supplement for Borinca, I., Vincenzo, I., and Valsecchi, G. (2020). Men’s discomfort and anticipated sexual misclassification due to counter-stereotypical behaviors: The interplay between traditional masculinity norms and perceived men’s femininization. *Sex Roles.* University of Geneva. Email: Islam.Borinca@unige.ch

***Norm manipulation (Experiment 2)***

***Feminizations Condition***

**
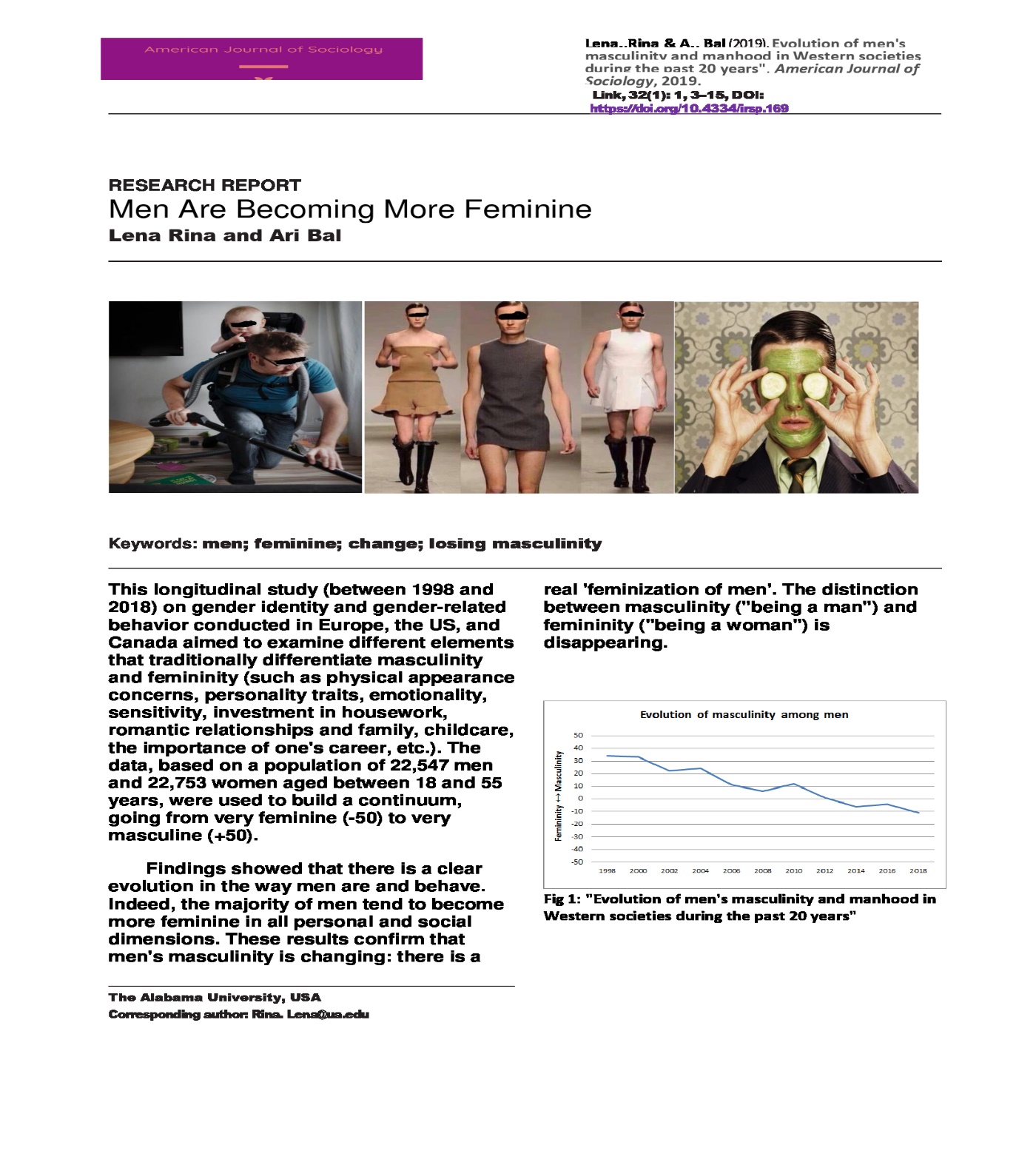
**

***Control condition***

No information was provided in the control condition.

***Norm manipulation (Experiment 3) in the Albanian language***

*General information (provided in both conditions)*

Ne do ju prezentojmë një përmbledhje të rezultateve nga një studim ndër vite në lidhje me identitetin gjinor dhe sjelljet e mundshme gjinore të realizuara në mes viteve 1998 dhe 2018 në shtetet perëndimore (Shtetet Perëndimore Evropiane, Amerikë, Kanadë, Australi etj). Studimi kishte të bënte me elementet e ndryshme që tradicionalisht e dallojn të qenurit mashkull (pra maskulizmin) dhe feminitetin (të qenurit femërorë; si paraqitja fizike, ana emocionale, ndjeshmëria, investimi në punët e shtëpisë (larje e enëve, gatimi), lidhja romantike dhe familja, përkujdesi ndaj fëmijëve (pastrimi i fëmijëve të vegjël) si dhe ndaj të tjerëve etj). Të dhënat e mbledhura deri më tani, bazuar në një popullatë prej 22,547 meshkuj dhe 22,753 femra të moshës 18 - 55 vjeçare, ishin përdorur për të ndërtuar një kontinium (vazhdimsi) e cila shkon nga të qenuritë shumë femërorë (-50) deri tek të qenuritë shumë mashkullor (+50).

*Masculine norm condition*

Siç shihet në figurën e mëposhtme, ky studim tregon/rezulton se në përgjithsi nuk ka ndryshueshmëri në mënyrën sesi meshkujt janë/duken apo sillen. Në fakt, shumica e meshkujve tentojnë të qëndrojnë sa më shumë mashkullor/burrëror në të gjitha aspektet personale dhe sociale. Rezultatet e këtij studimi konfirmojnë se të qenuritë burrë/mashkull është diçka stabile : **meshkujt janë meshkuj/burra dhe dallojnë prej femrave**. Dallimi në mes të qenuritë ***mashkullor ("të qenuritë burrë" ) dhe feminitetit ("të qenurit femër") është diçka thelbësore e cila qëndron akoma.***


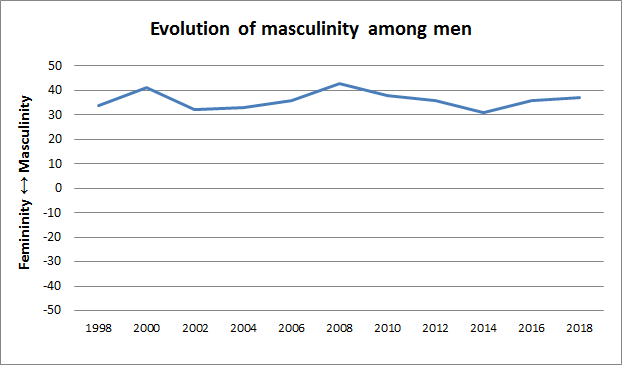


Burimi: " Evoluimi i të qenuritë mashkull dhe burrëria në shoqërinë perëndimore në 20 vitet e fundit" Journali Amerikan i Sociologjisë, 2019.

*Feminization norm condition*

Siç shihet në figurën e mëposhtme, ky studim tregon/rezulton se në përgjithsi se është një evolucion/ndryshim në mënyrën se si meshkujt janë dhe sillen. Në fakt, shumica e meshkujve po tentojnë që të feminizohen në gjitha aspektet personale dhe sociale. Rezultatet e këtij studimi konfirmojnë se të qenuritë mashkull po ndryshon: po ekziston një lloj 'feminizimi i meshkujve'. **Dallimi në mes maskulinizmit ("të qenuritë mashkull") dhe feminitetit (" të qenuritë femër") është në zhdukje e sipër**.


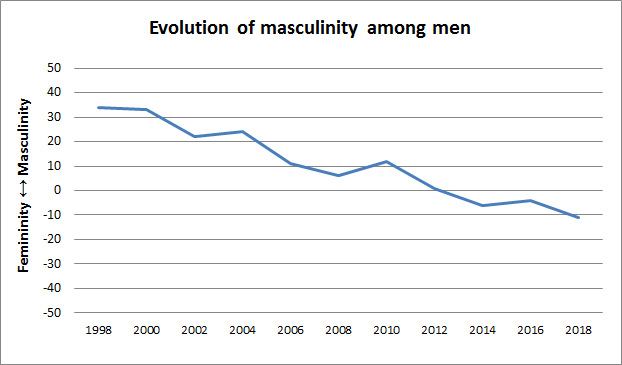


Burimi: " Evoluimi i të qenuritë mashkull dhe burrëria në shoqërinë perëndimore në 20 vitet e fundit" Journali Amerikan i Sociologjisë, 2019.

**Items for Likelihood of being misclassified in the Albanian version**.

**Ju lutemi përgjigjuni pyetjeve në vijim:**

|  | **Nuk ka gjasa** | ⇐ | | ⇔ | ⇒ | | **Ka shumë gjasa** |
| --- | --- | --- | --- | --- | --- | --- | --- |
| Sa mendoni që do kishte gjasa që dikush që nuk ju njeh juve automatikisht do të mendonte se ju jeni gej/homoseksual nëse ai apo ajo do ju shihte juve duke i bërë këto sjellje? | 1 | 2 | 3 | 4 | 5 | 6 | 7 |
| Sa do të kishte gjasa që një person heteroseksual automatikisht do të mendonte se ju jeni gej/homoseksual nëse ai apo ajo do ju shihte juve duke i bërë këto sjellje? | 1 | 2 | 3 | 4 | 5 | 6 | 7 |
| Sa do të kishte gjasa që një vajzë/femër automatikisht do të mendonte se ju jeni gej/homoseksual nëse ajo do ju shihte juve duke i bërë këto sjellje? | 1 | 2 | 3 | 4 | 5 | 6 | 7 |
